# Factors Influencing Microbiological Biodiversity of Human Foot Skin

**DOI:** 10.3390/ijerph16183503

**Published:** 2019-09-19

**Authors:** Aleksandra Steglińska, Anita Jachowicz, Justyna Szulc, Justyna Adamiak, Anna Otlewska, Katarzyna Pielech-Przybylska, Beata Gutarowska

**Affiliations:** Institute of Fermentation Technology and Microbiology, Lodz University of Technology, 90-924 Łódź, Poland

**Keywords:** microorganisms, foot skin, biodiversity, high-throughput sequencing

## Abstract

The aim of the study was to analyze the microbiological biodiversity of human foot skin with respect to factors such as age, gender, frequency of foot washing and physical activity, and to select indicator species to be considered when designing textile materials with antimicrobial properties used for sock and insole production. The experiment was carried out on a group of 40 people. The number of microorganisms was determined using culture-dependent methods. Biodiversity was determined using culture followed by genetic identification based on 16S rRNA gene sequencing (bacteria), ITS region (fungi), or using Illumina next-generation sequencing (in a group of eight selected individuals). The total bacterial number on women’s feet was on average 1.0 × 10^6^ CFU/cm^2^, and was not statistically significantly different than that of men’s feet (mean 1.2 × 10^5^ CFU/cm^2^). The number of bacteria, in most cases, decreased with age and with increased frequency of physical activity. The number of bacteria increased with diminishing feet-washing frequency; however, statistically significant differences were found between groups. The number of fungi was not significantly different amongst groups. Bacteria belonging to the phyla Firmicutes, Proteobacteria and Actinobacteria constituted the main microorganisms of the foot skin. *Ascomycota* and *Basidiomycota* predominated amongst the fungi. The presence of specific species varied in groups depending on the factors mentioned above. Two of the species identified were classified as pathogens (*Neisseria flavescens* and *Aspergillus fumigatus*). These findings suggest that it is necessary to extend the list of microorganisms tested on textiles with respect to hygienic properties.

## 1. Introduction

Human skin is a complex and dynamically changing environment colonized by various microorganisms [1] that have adapted to the adverse conditions present on its surface. These include slightly acidic pH, the presence of antimicrobial peptides and unsaturated fatty acids as well as constant peeling of the epidermis [2,3]. On a 1 cm^2^ surface area of human skin, there are about 10^6^ microbes [4], with the number and type varying depending on the area of occurrence. Bacteria of the genus *Propionibacterium* and *Staphylococcus* dominate sebaceous areas (forehead). Moist areas (feet) harbor *Corynebacterium* and *Staphylococcus*, while there is a great variety of bacteria with the predominance of β-Proteobacteria and Flavobacteriales in dry areas (elbows) [5].

It is estimated that fewer than 10% of microorganisms occurring in natural ecosystems can be identified in laboratory conditions using traditional culture techniques. Most microorganisms remain unculturable or hard-to-culture because they require unique factors that are difficult to create in vitro. In addition, the number of easily- and rapidly-growing microorganisms may be overestimated. Therefore, these methods do not reflect the actual microbiome composition. Only recently, the development of culture-independent molecular techniques has enabled the compilation of an increasingly reliable description of the human skin microbiome [1,2]; it is dominated by only four bacterial phyla: Actinobacteria (genera *Propionibacterium*, *Corynebacterium*), Firmicutes (genera *Staphylococcus*, *Streptococcus*), Proteobacteria (genera *Pseudomonas*, *Acinetobacter* and *Janthinobacterium*) and Bacteroidetes (*Sphingobacterium* and *Chryseobacterium*) [5,6,7,8]. Amongst the fungi, representatives of both *Ascomycota* and *Basidiomycota* phyla are considered to be normal microorganisms on human skin. The lipophilic *Malassezia* genus dominates the skin of the trunk and hands, while the skin of the feet is colonized by genera such as *Aspergillus*, *Cryptococcus*, *Epicoccum*, *Rhodotorula*, *Candida*, *Trichosporon* and others [9,10].

Many of the microorganisms isolated from skin are benign human commensals, while some may become pathogenic. Nevertheless, it has to be remembered that the division of microorganisms occurring on human skin into those that are harmless and those that constitute pathogens depends to a large extent on the organism’s ability to resist infection and on the properties of a microorganism [11].

People are born from substantially sterile environments, and they quickly become colonized by microorganisms. The kind of colonizing microorganisms depends on the delivery method (natural or caesarean section) [12]. The first years of life are crucial for the shaping of human skin microorganisms that is unstable in this period [13]. In subsequent years, the microbiome undergoes changes depending on various factors. These include, age, gender, immune system medications (e.g., steroids, antibiotics) and diseases (e.g., diabetes), as well as the environment—climate, exposure to ultraviolet radiation, ethnicity and lifestyle—hygiene, the nature of the work performed, type of clothing worn, diet and smoking tobacco products [1,14,15]. The differences in skin pH, hormone, sebum and sweat production as well as lifestyle differences are believed to provide an explanation for the variability in the microbiome of the human skin resulting from age and gender differences. A greater diversity of microorganisms is observed in women who more often use cosmetics that may disturb the structure of microorganism populations residing on the skin [1,7]. Washing, widely considered the most effective means of preventing infections, has an influence on the skin microbiome. Research shows, however, that although this action removes transient microorganisms, the total number of microorganisms remains at a similar level and can even increase immediately after washing due to the spread of microorganisms trapped in skin pores. The influence of washing and its frequency on the skin microbiome requires further research [1,16]. Engagement in sports, affecting the production of sweat promoting the development of microorganisms, may be a factor that influences the occurrence of microorganisms on the surface of foot skin.

An important factor limiting the development of microorganisms on foot skin is the use of fabrics containing biocides with hygienic and antimicrobial properties. Such fabrics are used in the textile industry for the production of, among others, socks, insoles, mattresses and tights. When modeling nonwoven fabrics to obtain antimicrobial properties, one should consider the inhibition of the growth of commensal microorganisms constantly present on the skin, the growth of which promotes the unpleasant odor of sweat, and bactericidal activity against pathogenic species only. At present, the antimicrobial activity of functional fabrics is not evaluated for naturally occurring microorganisms on the skin, but only for selected pathogens. There is therefore a need to determine the species of microorganisms on which analysis of the hygienic activity of functional fabrics should be conducted, bearing in mind age groups, gender and physical activity, with respect to the use of the nonwoven fabric.

The aim of the present work was to analyze the microbial biodiversity of human foot skin, to determine the impact of factors such as age, gender, foot washing and frequency of physical activity. We also compared results obtained by culture and a high-throughput sequencing method to identify indicator species to be considered when designing materials with antimicrobial properties that are used in sock and footwear insole production.

## 2. Materials and Methods

### 2.1. Characteristics of the Study Participants

The analysis of the microbiological biodiversity of the human foot skin was performed on a group of 40 volunteers chosen according to age and gender, so as to achieve four age groups (0–10, 11–17, 18–50 and >60 years), each composed of ten individuals (five males and five females). All volunteers (the legal guardian for persons <18 years). All subjects gave their informed consent for inclusion before they participated in the study. The study was conducted in accordance with the Declaration of Helsinki, and the protocol was approved by the Ethics Committee of 07/2019. Subjects filled in anonymized forms, on the basis of which they were further stratified according to factors such as feet-washing frequency (once/twice a day, every other day, once a week) and physical activity frequency (three or more times a week, 1–2 times per week, no activity). Among research participants: 28 people (70%) washing feet once a day, six (15%) twice a day and six (15%) every other day, 11 participants (27.5%) exercising 1–2 times per week, seven (17.5%) three times per week and above and 22 (55%) declared lack of physical activity

### 2.2. Sampling

Samples for microbiological testing were taken by swabbing the whole sole of the foot. Foot skin swabs were performed in the evening, immediately before washing. Therefore, the interval between washing and sampling was approximately 12 h, depending on the frequency of cleaning the foot. Swabs were placed in plastic tubes containing physiological saline solution: From one foot we obtained a sample for quantitative analysis and identification of cultured microorganisms through sequencing of 16S rRNA for bacteria and ITS region for fungi; from the other foot we obtained a sample for identification of microorganisms using Illumina next-generation sequencing. Microbe suspensions were recovered from the swabs by shaking them for 5 min. The lengths and the widths of the feet of every participant were measured in order to calculate the surface area for CFU/cm^2^ calculation.

### 2.3. Culture Method

The general numbers of cultured bacteria and fungi in samples taken from feet were determined using the surface plating method. Following triplicate sampling from the original suspension, a series of dilutions in physiological saline solution (0.85% NaCl) were performed and plated onto tryptic soy agar medium (TSA, Merck, Germany) with nystatin (0.3%) for bacteria, and onto malt extract agar medium (MEA, Merck, Germany) with chloramphenicol (0.1%) for fungi. Samples were incubated at 30 °C ± 2 °C for 24–48 h (bacteria) or 3–5 days (fungi). Following the incubation, colonies were counted and the results obtained were expressed as CFU/cm^2^.

Pure cultures of bacteria and fungi were characterized macroscopically. Bacterial strains were Gram-stained. Subsequently, we performed genetic identification of bacteria based on 16S rRNA sequencing using 27f/1492r universal primers [17] and the ITS region in yeast using ITS1/ITS4 primers [18]. Genomic DNA was extracted from bacteria using the Genomic Mini kit (A & A Biotechnology, Gdynia, Poland), and from fungi using the Plant & Fungi DNA Purification Kit (Eurx, Gdańsk, Poland). The 25-µL reaction mix contained RedTaq Ready Mix polymerase (1.5 U), primers (40 pmol), a DNA template (20 ng) and high-purity water. PCR reactions were performed on a MJ Mini Gradient Thermal Cycler (Bio-Rad, Hercules, CA, USA). PCR reaction products were analyzed by electrophoresis on a 1% (*w*/*v*) agarose gel using a 100-bp DNA ladder (Eurx, Gdańsk, Poland). Sequencing reactions were performed using the BigDye^®^ Terminator v 3.1 kit (Applied Biosystems, Life Technologies, Carlsbad, CA, USA). Sequencing reaction products were separated on a 3730xl DNA analyzer capillary sequencer. Nucleotide sequences were compared to sequences available in the National Center for Biotechnology Information the BLAST 2.2.30+ tool [19]. The identification of filamentous fungi was performed on the basis of macro- and microscopic observation of strains seeded onto MEA (Malt Extract Agar, Merck, Darmstadt, Germany) and Czapek yeast extract agar (CYA, Difco, Franklin Lakes, NJ, USA) media, using taxonomic keys [20,21].

### 2.4. DNA Extraction and High-Throughput Sequencing.

Next-generation sequencing utilizing Illumina technology was used to identify unculturable bacteria and fungi (molds and yeast) isolated from the foot surface. The complete genomic DNA was extracted from the swabs using the kit for DNA purification from swab samples (no. 025-25, A & A Biotechnology, Poland) based on chemical lysis. DNA extraction was performed according to manufacturer’s instructions except for the following modification to the first step: Swabs were disrupted by bead-beating for 1 min employing beads matrix (1.4 mm ceramic spheres, 0.1 mm silica spheres and one 4 mm glass bead). The concentration of the isolated DNA was measured using a Qubit 2.0 Fluorometer (Invitrogen/Life Technologies, Carlsbad, CA, USA).

The amplification of the V3-V4 region of the 16S rRNA gene was performed using 341F and 785R universal primers [22], whereas those of the ITS region in yeast used ITS1/ITS2 primers [18]. All PCR reactions were carried out in 25 µL volume containing 12.5 µL Q5 Hotstart High-Fidelity 2x Master Mix (New England Biolabs, MA, USA), 5 µL of each primer (1 µM) and 2.5 µL of DNA as template (10–20 ng). To exclude the possibility of cross-contamination, a negative control (no DNA template) was included in each PCR reaction. Amplifications for both bacteria and fungi were performed applying cycling conditions consisting of an initial denaturation (95 °C, 3 min), followed by 25 cycles as follows: Denaturation (95 °C, 30 s), primer annealing (55 °C, 30 s), elongation (72 °C, 30 s) and a final DNA elongation (72 °C, 5 min). PCR reaction products were purified using the AMPure kit (Agencourt Bioscience Corporation, Beverly, MA, USA), and were then used as templates in reaction-containing adapter sequences. Bar-coded PCR products were sequenced using an Illumina MiSeq sequencer (Illumina, Inc. San Diego, CA, USA).

Gene libraries were subjected to bioinformatic analysis using QIIME version 1.8.0 [23]. The raw reads were demultiplexed and quality-filtered on the MiSeq instrument using the MiSeq Reporter (MSR) v2.4 (BaseSpace) program (Illumina, Inc. San Diego, CA, USA). Nucleotide sequences were clustered based on 97% of similarity. Taxonomy was determined using the Greengenes database for bacteria [24], and the UNITE database for fungi [25].

### 2.5. Mathematical and Statistical Analyses

The arithmetic means, standard deviations of the microorganism number isolated from the foot skin and frequencies of the microorganisms occurrence were calculated using Microsoft^®^ Excel 2010 (Redmond, DC, USA). Frequencies of the occurrence were calculated according to Equation (1). To evaluate the differences between microorganism number on human foot skin with respect to age group, washing frequency and physical activity the Kruskal–Wallis nonparametric test was used followed by the Mann–Whitney U-test (at significance level α = 0.05). All analyses were conducted using STATISTICA 10 software (Tibco Software, Palo Alto, CA, USA).

f = (a/N) × 100%(1)

f—microorganisms isolation frequency; a—number of examined people from whom the given strain of bacteria or fungi was isolated within the tested group; N—total number of examined people in the tested group.

## 3. Results and Discussion

### 3.1. The Influence of the Studied Factors on the Number of Bacteria and Fungi on Foot Skin

The overall microorganism number on foot surfaces of the studied persons totaled on average 5.0 × 10^5^ CFU/cm^2^ (bacteria) and 4.7 × 10^1^ CFU/cm^2^ (fungi) and varied with respect to age, gender and microorganism type. The range reported by Grice et al. [4] was 1.0 × 10^4^ to 1.0 × 10^6^ bacteria/cm^2^ on the skin of the inner elbow (a high-humidity site, similar to feet). The results obtained in the current experiment showed that the percentage of fungi in the pool of all isolated species was low and comprised only 0.1%. Oh et al. [26] reported that fungi constituted a higher percentage in the pool of all microorganisms, ranging from 0.3% (heel) to 0.7% (interdigital spaces).

The mean number of bacteria on the feet of women was 1.0 × 10^6^ CFU/cm^2^ and was higher than that of men by an order of magnitude (1.2 × 10^5^ CFU/cm^2^) however these differences were not statistically significant (*p* > 0.05). A similar relationship was noted for fungi; the average fungi number equaled 8.1 × 10^1^ CFU/cm^2^ in women and 1.6 × 10^1^ CFU/cm^2^ in men (Table 1) It has been shown statistically significant differences in the number of fungi in women and men tested groups (*p* < 0.05). With increasing age, the number of bacteria in both women and men statistically significant decreased, from an average of 3.9 × 10^6^ (girls, 0–10 years) and 3.6 × 10^5^ CFU/cm^2^ (boys, 0–10 years) to an average of 1.1 × 10^4^ (women, >60 years) and 7.6 × 10^3^ CFU/cm^2^ (men, >60 years; *p* < 0.05). The highest overall number of fungi was found in the group of women over 60 years of age (mean 2.1 × 10^2^ CFU/cm^2^) and the lowest was in men aged 18–50 (average <1 CFU/cm^2^; Table 1).

Bacteria number statistically significant increased with decreasing feet washing frequency and ranged on average from 8.8 × 10^3^ (twice daily) to 1.1 × 10^6^ CFU/cm^2^ (every other day; *p* < 0.05). The overall fungi number was at a similar level independently of the frequency of feet washing as confirmed by statistical analysis and ranged on average from 5.4 × 10^1^ (twice daily) to 5.6 × 10^1^ CFU/cm^2^ (once daily; Table 2).

A decrease in the overall bacteria number on the foot surface was observed along with an increase in physical activity from the level of 6.9 × 10^5^ (no physical activity) to 2.2 × 10^4^ CFU/cm^2^ (three times and above); however, no statistically significant differences were shown between groups. The highest overall fungi number was ascertained in the group of people who undertook physical activity 1–2 times a week (5.7 × 10^1^ CFU/cm^2^ on average) and the statistically significant lowest in people working out three or more times a week (1.2 × 10^1^ CFU/cm^2^ on average; *p* < 0.05). No significant correlation was noted between group with lack of physical activity and practicing sport regardless of its frequency (*p* > 0.05; Table 2).

### 3.2. Determining the Influence of the Studied Factors on the Microbiological Biodiversity of Foot Skin Using Culture Method

The frequency of species on foot skin identified by culture and by genetic (16S RNA and ITS) methods ranged from 2.5–90% for bacteria, and from 2.5–25% for fungi (Table 3).

*Staphylococcus haemolyticus* (90%), *Staphylococcus hominis* (52.5%) and *Micrococcus luteus* (22.5%) were the most frequently isolated bacteria. These results are confirmed by the studies by Costello et al. [8], who showed that the surface of foot skin is mainly colonized by bacteria of the *Staphylococcus* genus. The filamentous fungi most frequently isolated were *Aspergillus fumigatus* (25%), *Penicillium glabrum* (17.5%), *Aspergillus candidus* (12.5%) and *Aspergillus niger* (12.5%), whereas *Naganishia diffluens* (17.5%) and *Wickerhamomyces anomalus* (15%) dominated amongst yeasts (Table 3). Findley et al. [10] showed that human foot skin surface was the site with the highest biodiversity of fungal species represented by genera *Aspergillus*, *Cryptococcus*, *Malassezia*, *Rhodotorula*, *Epicoccum*, among others.

**Figure 1:**
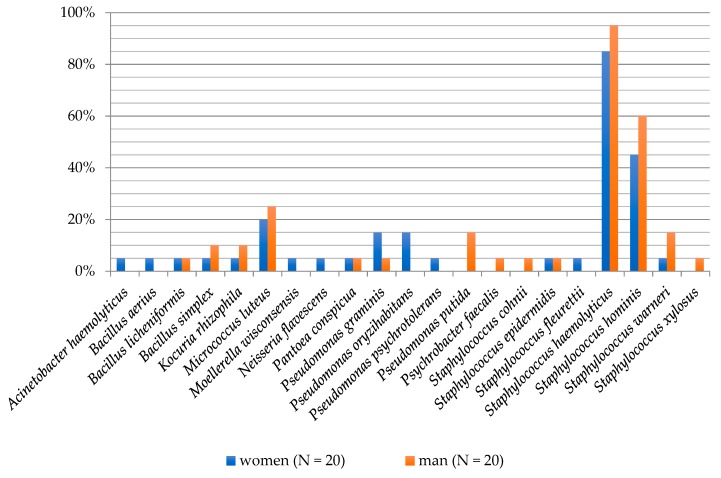
Frequency of the occurrence of bacteria on the foot skin depending on the gender of examined people.

A higher biodiversity of cultured bacterial species on foot skin was observed in women (17 individual species) than in men (14 individual species). Such correlation is consistent with results obtained by Fierer et al. [7], who used hands of subjects for testing. In the present study, *Staphylococcus haemolyticus* (85%), *Staphylococcus hominis* (45%), *Micrococcus luteus* (25%) and bacteria from the *Pseudomonas* genera: *P. graminis* and *P. oryzihabitans* (15% each) were isolated with the highest frequency on women’s feet. Men’s feet were dominated by *S. haemolyticus* (95%), *S. hominis* (60%), *M. luteus* (25%) and *Staphylococcus warneri*, and *Pseudomonas putida* (15% each; Figure 1).

**Figure 2:**
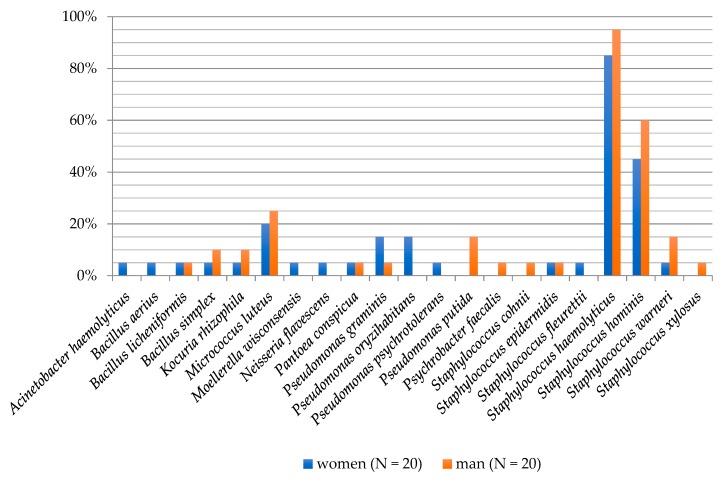
Frequency of the occurrence of fungi on the foot skin depending on gender of examined people.

A higher biodiversity of cultured fungi was noted in men (19 species) than in women (17 species). Women’s feet were dominated by *Aspergillus fumigatus* (30%), *Penicillium glabrum* (30%) and *Aspergillus candidus* (20%) molds, and *Naganishia diffluens* (20%) and *Wickerhamomyces anomalus* (20%) yeasts. *Aspergillus fumigatus* (20%) was the mold most frequently isolated from men’s feet (Figure 2).

**Figure 3:**
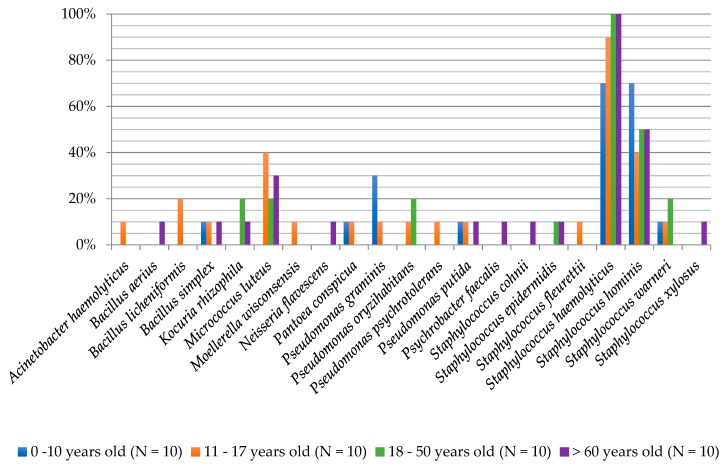
Frequency of the occurrence of bacteria on the foot skin depending on the age of examined people.

The degree of biodiversity of cultured bacterial species in the individual age groups was as follows: 0–10 years (six species), 11–17 years (14 species), 18–50 years (seven species) and >60 years (12 species). In the group of children aged 0–10, *S. haemolyticus* and *S. hominis* were isolated with the highest frequency (70%), while *P. graminis* bacteria had a frequency of 30%. Bacteria from the *M. luteus* species were present in all age groups at a frequency of 20–40%, except for children aged 0–10 years. *S. haemolyticus* (90%), *S. hominis* (40%), *M. luteus* (40%) and *Bacillus licheniformis* (20%) species dominated in teenagers aged group 11–17 years. *S. haemolyticus* species occurred at a 100% frequency in adults (18–50 years) and in the elderly (>60 years). *S. hominis* bacteria were isolated in both groups at a frequency of 50%. Moreover, in the 18–50 year group, *Kocuria rhizophila*, *M. luteus*, *P. oryzihabitans* and *S. warneri* were present in 20% (Figure 3).

**Figure 4:**
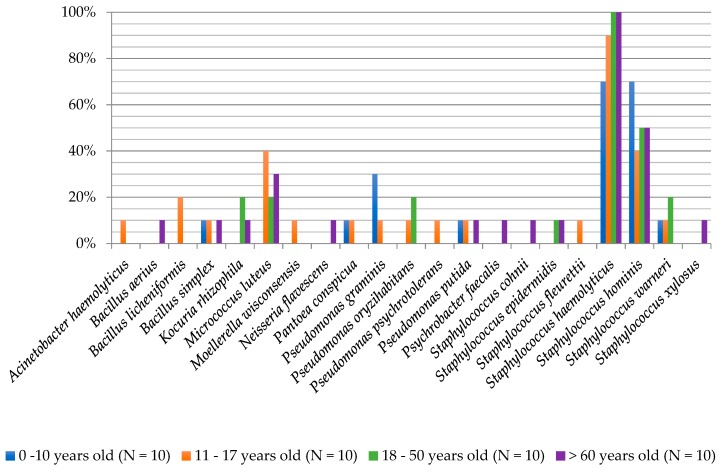
Frequency of the occurrence of fungi on the foot skin depending on the age of examined people.

The degree of biodiversity of cultured fungi in the individual age groups was as follows: 0–10 years (ten species), 11–17 years (12 species), 18–50 years (seven species) and >60 years (13 species). These results mirror the studies of Jo et al. (2016), who showed higher biodiversity in children up to 14 years of age than in adults (20–30 years) [27]. In the 0–10 year group, *A. fumigatus* (50%), *P. glabrum* (40%) and *Rhodotorula mucilaginosa* (30%) were isolated with the highest frequency. Amongst people aged 11–17 years, *W. anomalus* (40%) and *Aspergillus candidus* (30%) dominated, while *Aspergillus flavus*, *A. fumigatus*, *Aspergillus niger*, *Penicillium citrinum*, *Meyerozyma quilliermondii*, *N. diffluens* and *Pichia kudriavzevii* were present in 20% of subjects. In the group of participants aged 18–50 years, *M. quilliermondii* (30%) as well as *P. glabrum* (20%) and *N. diffluens* (20%) were isolated most frequently. In the group of persons >60 years of age, molds from the *Aspergillus* genus: *A. candidus*, *A. niger*, *A. fumigatus* and from the *Penicillium* sp. genus (2) and *Geotrichum candidum* species were isolated at the highest frequencies (20%; Figure 4).

**Figure 5:**
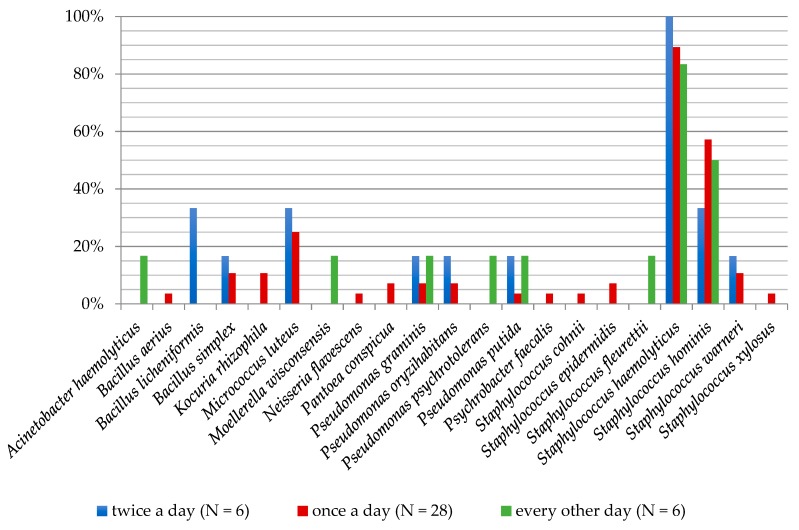
Frequency of the occurrence of bacteria on the foot skin depending on the washing feet frequency of examined people.

The highest biodiversity of cultured bacteria was noted in the group who washed feet once daily (16 species), followed by people washing feet twice daily (nine species) and the lowest in the variant of feet washing every other day (eight species). Amongst people washing feet twice daily, *S. haemolyticus* species occurred at a 100% frequency. Additionally, *B. licheniformis*, *M. luteus* and *S. hominis* were isolated at a frequency of 33.33%. In the group of people washing feet once a day, the presence of *S. haemolyticus* (89.29%), *S. hominis* (57.14%) and *M. luteus* (25%) was noted at the highest frequencies. People washing feet every other day were dominated by *S. haemolyticus* (83.33%) and *S. hominis* (50%; Figure 5).

**Figure 6:**
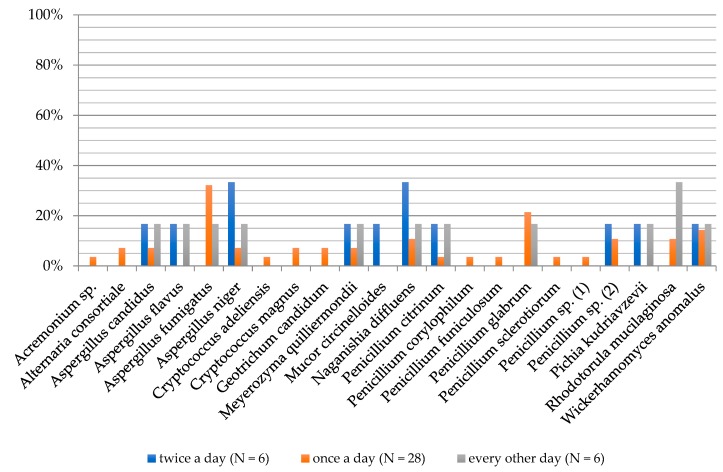
Frequency of the occurrence of fungi on the foot skin depending on the washing feet frequency of examined people.

The highest degree of biodiversity of fungal species was found in the group of people washing feet once daily (19 species), followed by those washing feet every other day (11 species). It was lowest in the group of people washing feet twice a day (ten species). *A. niger* and *N. diffluens* (33.33% each) dominated amongst people washing feet twice daily. *A. fumigatus* (32.14%) and *P. glabrum* (21.43%) species were on feet of people washing once a day. In the group of people washing feet every other day, *Rhodotorula mucilaginosa* (33.33%) yeast was isolated with the highest frequency (Figure 6).

**Figure 7:**
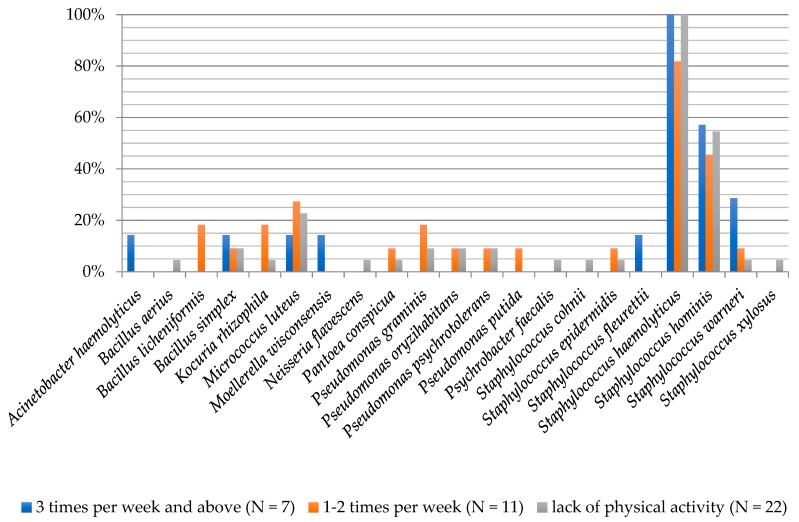
Frequency of the occurrence of bacteria on the foot skin depending on the physical activity frequency of examined people.

The degree of biodiversity of bacterial species in groups of participants divided according to physical activity was shaped by the following: Physical activity three or more times a week, eight species; 1–2 times a week, 13 species and no physical activity, 16 bacterial species. Amongst people working out three times a week, *Staphylococcus haemolyticus* species was present at a frequency of 100%, *S. hominis* (57.14%) and *S. warneri* (28.57%) were also present at a high frequency. People exercising 1–2 times a week were dominated by *S. haemolyticus* (81.82%), *S. hominis* (45.45%) and *M. luteus* (27.27%). In people who undertook no physical activity, *S. haemolyticus* and *S. hominis* were isolated at 100% frequency, in 27.27% of the samples there was also *M. luteus* (Figure 7).

**Figure 8:**
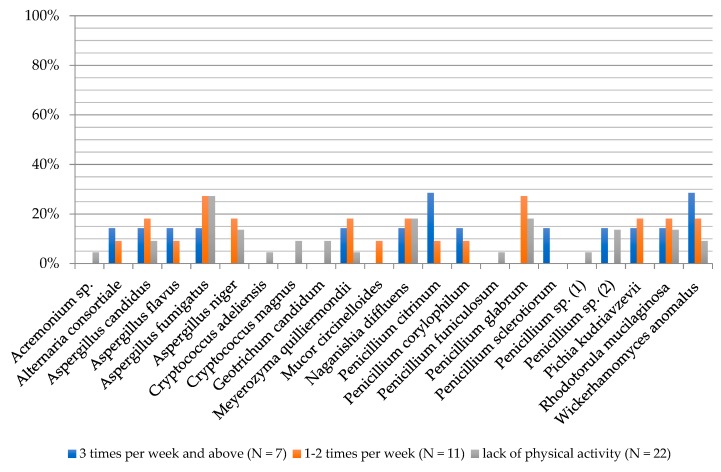
Frequency of the occurrence of fungi on the surface of human feet skin depending on the physical activity frequency of examined people.

The biodiversity of fungal species was lowest in people working out three or more times a week (13 species). In the remaining groups, the values were as follows: 15 individual species, no physical activity and 14 individual species, activity 1–2 times a week. Amongst people working out at least three times a week, *P. citrinum* (28.57%) and *W. anomalus* (28.57%) dominated. In the group of people exercising 1–2 times a week, *A. fumigatus* (27.27%) and *P. glabrum* (27.27%) molds were present at the highest frequency, and in the group of people who undertook no physical activity one species dominated: *A. fumigatus* (27.27%; Figure 8).

### 3.3. Microbiological Biodiversity of Foot Skin on the Basis of High-Throughput Sequencing

**Figure 9:**
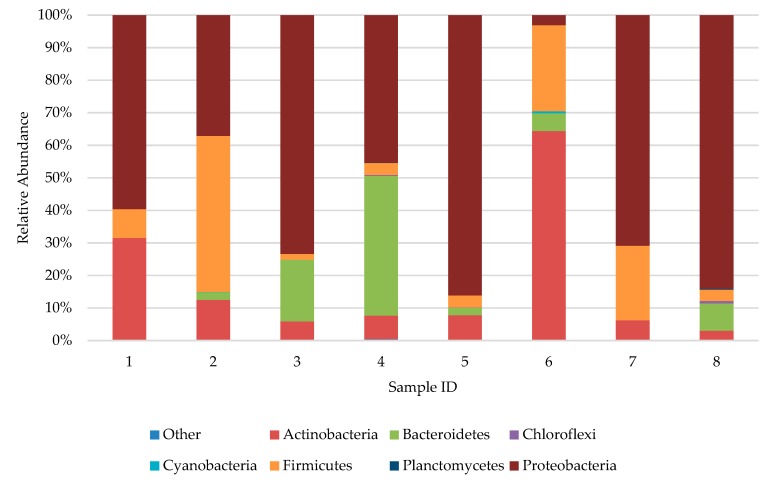
Biodiversity of bacteria in samples acquired from feet skin of examined people. 1—man, 0–10 years old; 2—woman, 0–10 years old; 3—man, 11–17 years old; 4—woman, 11–17 years old; 5—man, 18–50 years old; 6—woman, 18–50 years old; 7—man, >60 years old; 8—woman, >60 years old.

Selected samples, two for each age group (one male and one female) were subjected to high-throughput sequencing on the Illumina platform. Amongst selected men (samples 1, 3, 5 and 7; Figure 9), bacteria from phylum Proteobacteria dominated (from 59.61% in sample 1–86.12% in sample 5) and amongst women (samples 2, 4, 6 and 8; Figure 9) the distribution varied. In samples 4 and 8, bacteria from phylum Proteobacteria dominated as well (45.39% and 84.11%, respectively), in sample 2 it was Firmicutes (47.88%) and in sample 6 it was Actinobacteria (64.39%). Bacteria from the Proteobacteria phylum dominated in the samples taken from persons aged 11–17 and >60 years of age. It is worth highlighting that the relative number of Bacteroidetes phylum was at a significant level only in the 11–17 year group (18.92% and 42.90%); in the remaining groups it did not exceed 10% (Figure 9). The analysis of analogous samples performed by culture method showed instead, that the Firmicutes phylum constituted the predominant bacterial species independent of age or gender. The studies of Capone et al. [13] showed that children’s skin is colonized mainly by bacteria from Firmicutes, followed by Actinobacteria, Proteobacteria and Bacteroidetes. In the case of adults, the order was as follows: Proteobacteria, Actinobacteria and Firmicutes. These results overlap partially with the ones obtained in the present study. In three out of four samples taken from adults >18 years of age, high-throughput sequencing confirmed the predominance of Proteobacteria, whilst the subsequent order varied depending on the sample. For children aged 0–10 years, one out of two samples were dominated by Firmicutes, as similar to earlier studies.

**Figure 10:**
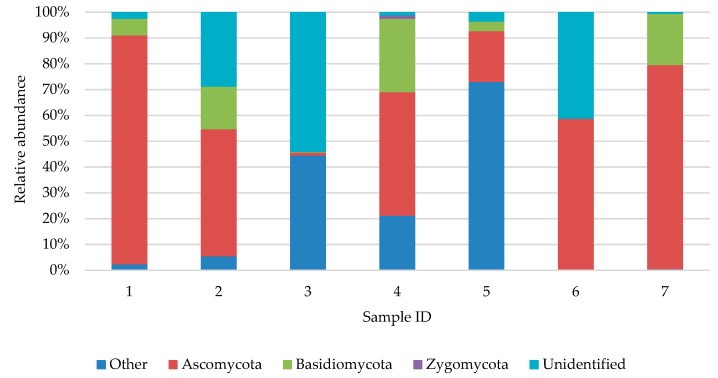
Biodiversity of fungi in samples acquired from feet skin of examined people. 1—man, 0–10 years old; 2—woman, 0–10 years old; 3—man, 11–17 years old; 4—woman, 11–17 years old; 5—man, 18–50 years old; 6—woman, 18–50 years old; 7—man, >60 years old.

Amongst the selected women (samples 2, 4 and 6; Figure 10), fungi of Ascomycota dominated in all samples (from 47.84–58.60%). This was confirmed by analyzing analogous samples by culture. Amongst men (samples 1, 3, 5 and 7; Figure 10) the partition varied; in samples 1 and 7, the predominance of Ascomycota phylum was observed, in samples 3 and 5, unidentified species prevailed. However, detection by culture method indicated Ascomycota as the dominant phylum in three out of four analogous samples taken from men. In the age group 0–10 years, Ascomycota phylum dominated (49.27% and 88.67%; Figure 10).

### 3.4. Comparison of Culture and High-Throughput Sequencing Methods

Microorganism identification on feet (in samples 1–8; Table 4) by culture showed the presence of 12 bacterial species (21 species in all 40 subjects) and 15 fungal species (23 species in all 40 subjects), whereas the use of high-throughput sequencing in analogous samples helped identify species or phyla from 106 bacterial and 41 fungal strains (Tables S1 and S2). Both methods confirmed the occurrence of, amongst others, bacteria from the *Staphylococcus*, *Pseudomonas* and *Micrococcus* genera and fungi from *Penicillium* and *Alternaria* genera. Furthermore, the presence of bacteria from the *Moellerella* and *Kocuria* genera as well as fungi from the *Aspergillus, Geotrichum, Pichia, Cryptococcus* and *Naganishia* genera were detected only through culture. High-throughput sequencing method, on the other hand, helped detect bacteria from the *Corynebacterium* phylum that are difficult to culture in laboratory conditions in all samples (Table 4). This is in contrast to the findings of Fierer et al. [7] that indicated an 80% higher relative abundance of *Corynebacterium* on men’s hand skin than in women’s. The current study found that the average relative abundance of bacteria from *Corynebacterium* genus was slightly higher in women (Relative Abundance, RA = 2.70853) than in men (RA = 2.21903; Tables S1 and S2).

Two species amongst all those identified constitute category 2 threats, according to the Directive 2000/54/EC of the European Parliament and of the Council of 18 September 2000 on the protection of workers from risks related to exposure to biological agents at work [28]. These were *Aspergillus fumigatus* molds present in samples 2, 3 and 4, and *Neisseria flavescens* bacteria detected in sample 8 (Table 4). One has to bear in mind that *Actinomyces, Corynebacterium* and *Streptomyces* were identified at the level of genus by the high-throughput sequencing only, and may include pathogenic species.

According to Gao et al. [6] and Fierer et al. [7], bacteria from three phyla: Actinobacteria, Firmicutes and Proteobacteria, dominated on the skin of forearms and hands, respectively, as confirmed by the current experiments on human foot skin. Bacteria from the *Propionibacterium, Streptococcus, Staphylococcus* and *Corynebacterium* genera occurred in all or almost all samples analyzed in these studies. In a study on skin from the arm, Grice et al. [4], in turn, found that the majority of identified sequences belonged to Proteobacteria (mainly *Pseudomonas* and *Janthinobacterium*), followed by Actinobacteria (mainly *Corynebacterium, Kocuria, Propionibacterium, Microbacterium* and *Micrococcus*), Firmicutes (*Staphylococcus* and *Clostridium*) and Bacteroidetes (*Sphingobacterium* and *Chryseobacterium*). Nearly all of the mentioned genera (with the exception of *Clostridium*) were detected in the present study as well.

On the basis of the frequency of microorganism isolation and identification from foot skin using culture methods and high-throughput sequencing as well as potential pathogenicity, we classified species to be considered in studies of the design of textile products, i.e., socks and insoles endowed with antimicrobial properties (Table 5). Currently, for such an analysis, only pathogenic microorganisms from strain collections, e.g., *Staphylococcus aureus, Candida albicans, Scopulariopsis brevicaulis, Trichophyton mentagrophytes* and *Epidermophyton floccosum*, were used [29]. Such species rarely occur in the natural environment (as we and others showed). Moreover, authors of published reports proposed the inclusion of microorganisms such as *Escherichia coli, Pseudomonas aeruginosa, Staphylococcus epidermidis* and *Bacillus subtilis* into studies [30,31,32,33,34]. For this reason, based on present results when researching the antimicrobial activity of textile products, broadening of analysis by including 17 bacterial and 14 fugal strains, occurring as natural foot microorganisms should be considered (Table 5).

## 4. Conclusions

Human foot skin was colonized mainly by Proteobacteria, Firmicutes and Actinobacteria bacteria; *Ascomycota* and *Basidiomycota* fungi constituted only 0.01% of the microorganisms. Age and gender, as well as feet washing and sport practice frequency were factors that influenced the number and biodiversity of human foot skin microorganisms, The number of bacteria in women was on average an order of magnitude higher than that of men however these differences were not statistically significant. A bacteria number higher by almost two orders of magnitude was determined for the ages 0–10 years compared to >60; at 11–17 years, an increased contribution of Bacteroidetes phylum was detected. A higher biodiversity of bacteria and fungi occurred in females aged 11–17 and >60 than in males, in whom Proteobacteria bacteria dominated (similarly to the elderly). Bacteria number decreased with diminishing frequency of feet washing, while the greatest microorganism biodiversity was found in the group that washed feet once daily. A bacteria number and biodiversity does not depend on physical activity. In order to identify all microorganism species on foot skin combining culturing and high-throughput sequencing methods would reflect bacterial community composition more precisely than either of them can do alone. The detection of bacteria from *Corynebacterium* genus was shown only using the next generation sequencing method and *Moellerella, Kocuria* bacteria and *Aspergillus, Geotrichum, Cryptococcus*, *Pichia* and *Naganishia* fungi were found only using culture methods. Potential pathogens, *Aspergillus fumigatus, Neisseria flavescens* and species from *Actinomyces, Corynebacterium* and *Streptomyces* genera were detected on the foot skin of the study group (*N* = 40). Microbial species, which should be considered when designing socks and insoles with antimicrobial properties, were classified based on the frequency of isolation and identification in culture methods and high-throughput sequencing, as well as their potential pathogenicity.

## Figures and Tables

**Table 1:** Total number of bacteria and fungi depending on age and gender factors.

Microorganisms	Total Microorganisms Number (CFU/cm^2^)							
	Women				Man			
	0–10 Years Old	11–17 Years Old	18–50 Years Old	>60 Years Old	0–10 Years Old	11–17 Years Old	18–50 Years Old	>60 Years Old
Bacteria	X: 3.9 × 10^6 A^	X: 6.0 × 10^4 AB^	X: 1.2 × 10^4 B^	X: 1.1 × 10^4 B^	X: 3.6 × 10^5 A^	X: 1.0 × 10^5 AB^	X: 1.1 × 10^4 B^	X: 7.6 × 10^3 B^
	SD: 5.9 × 10^6^	SD: 7.7 × 10^4^	SD: 6.4 × 10^3^	SD: 8.5 × 10^3^	SD: 7.5 × 10^5^	SD: 2.1 × 10^5^	SD: 1.3 × 10^4^	SD: 8.8 × 10^3^
Total number of bacteria	X: 1.0 × 10^6 a^				X: 1.2 × 10^5 a^			
	SD: 3.2 × 10^6^				SD: 3.9 × 10^5^			
Fungi	X: 2.4 × 10^1 A^	X: 6.8 × 10^1 A^	X: 2.5 × 10^1 A^	X: 2.1 × 10^2 A^	X: 3.2 × 10^1 A^	X: 1.7 × 10^1 A^	X: <1	X: 1.5 × 10^1 A^
	SD: 3.5 × 10^1^	SD: 1.1 × 10^2^	SD: 3.3 × 10^1^	SD: 3.0 × 10^2^	SD: 2.9 × 10^1^	SD: 3.4 × 10^1^	SD: <1	SD: 3.1 × 10^1^
Total number of fungi	^2^ X: 8.1 × 10^1^				X: 1.6 × 10^1 b^			
	SD: 8.6 × 10^1^				SD: 2.8 × 10^1^			

X—mean; SD—standard deviation; A–B—means with the same letter are not significantly different (*p* < 0.05); a–b—means with the same letter are not significantly different, (*p* < 0.05).

**Table 2:** Total number of bacteria and fungi depending on washing feet and physical activity frequency factors.

Microorganisms	Total Microorganisms Number (CFU/cm^2^)					
	Washing Feet Frequency			Physical Activity Frequency		
	Twice a Day	Once a Day	Every Other Day	3 Times per Week and Above	1–2 Times per Week	Lack of Physical Activity
Bacteria	X: 8.8 × 10^3 A^	X: 5.6 × 10^5 A^	X: 1.1 × 10^6 B^	X: 2.2 × 10^4 A^	X: 6.4 × 10^5 B^	X: 6.9 × 10^5 A^
	SD: 6.1 × 10^3^	SD: 2.5 × 10^6^	SD: 2.5 × 10^6^	SD: 3.4 × 10^4^	SD: 1.9 × 10^6^	SD: 2.8 × 10^6^
Fungi	X: 5.4 × 10^1 A^	X: 5.6 × 10^1 A^	X: 5.5 × 10^1 A^	X: 1.2 × 10^1 A^	X: 5.7 × 10^1 B^	X: 5.6 × 10^1 AB^
	SD: 1.1 × 10^2^	SD: 1.4 × 10^2^	SD: 5.1 × 10^1^	SD: 2.5 × 10^1^	SD: 7.7 × 10^1^	SD: 1.6 × 10^2^

X—mean; SD—standard deviation A-B means (among groups of microorganisms) with the same letter are not significantly different (*p* < 0.05).

**Table 3:** Frequency of the microorganisms occurrence on foot skin.

Microorganisms Isolation Frequency Based on Samples Acquired from Examined People (*N* = 40)			
Bacteria	(%)	Fungi	(%)
*Acinetobacter haemolyticus*	2.5	*Acremonium* sp.	2.5
*Bacillus aerius*	2.5	*Alternaria consortiale*	5.0
*Bacillus licheniformis*	5.0	*Aspergillus candidus*	12.5
*Bacillus simplex*	7.5	*Aspergillus flavus*	5.0
*Kocuria rhizophila*	7.5	*Aspergillus fumigatus*	25.0
*Micrococcus luteus*	22.5	*Aspergillus niger*	12.5
*Moellerella wisconsensis*	2.5	*Cryptococcus adeliensis* *	2.5
*Neisseria flavescens*	2.5	*Cryptococcus magnus* *	5.0
*Pantoea conspicua*	5.0	*Geotrichum candidum*	5.0
*Pseudomonas graminis*	10.0	*Meyerozyma quilliermondii* *	12.5
*Pseudomonas oryzihabitans*	7.5	*Mucor circinelloides*	2.5
*Pseudomonas psychrotolerans*	2.5	*Naganishia diffluens* *	17.5
*Pseudomonas putida*	7.5	*Penicillium citrinum*	7.5
*Psychrobacter faecalis*	2.5	*Penicillium corylophilum*	5.0
*Staphylococcus cohnii*	2.5	*Penicillium funiculosum*	2.5
*Staphylococcus epidermidis*	5.0	*Penicillium glabrum*	17.5
*Staphylococcus fleurettii*	2.5	*Penicillium sclerotiorum*	2.5
*Staphylococcus haemolyticus*	90.0	*Penicillium* sp. (1)	2.5
*Staphylococcus hominis*	52.5	*Penicillium* sp. (2)	10.0
*Staphylococcus warneri*	10.0	*Pichia kudriavzevii* *	5.0
*Staphylococcus xylosus*	2.5	*Rhodotorula mucilaginosa* *	12.5
		*Wickerhamomyces anomalus* *	15.0

* yeast species; (1), (2)—strain no.

**Table 4:** Microorganisms identified by high-throughput sequencing and culture methods in samples acquired from feet skin of examined people.

Sample ID	Bacteria	Fungi
1	**Actinobacteria***: Brevibacterium, Oerskovia, Corynebacterium, Brachybacterium, Dietzia, Curtobacterium, Leucobacter, Microbacterium, Pseudoclavibacter, Salinibacterium, Yonghaparkia, Arthrobacter, Micrococcus, Sanguibacter, Rhodococcus, Streptomyces;***Bacteroidetes**: *Chryseobacterium;***Firmicutes**: Bacillus, Brochothrix, Paenibacillus, Sporosarcina, Paenisporosarcina, Exiguobacterium, Aerococcus, Helcococcus, Staphylococcus *, Facklamia, Carnobacterium;**Proteobacteria:***Devosia, Agrobacterium, Paracoccus, Achromobacter, Acidovorax, Psychrobacter, Pseudomonas, Phenylobacterium, Paracoccus, Pigmentiphaga, Ralstonia, Acinetobacter*	**Ascomycota*:*** Mycosphaerella, Arachnomyces, Penicillium *, Sagenomella; **Basidiomycota*:*** *Laetiporus, Goffeauzyma, Malassezia*
2	**Actinobacteria**: *Brevibacterium, Oerskovia, Corynebacterium, Brachybacterium, Dietzia, Rhodococcus, Streptomyces, Actinomyces, Curtobacterium, Leucobacter, Microbacterium, Pseudoclavibacter, Yonghaparkia, Arthrobacter, Micrococcus, Mycobacterium, Nocardioides, Nocardiopsis, Propionibacterium;***Bacteroidetes**: *Myroides, Chryseobacterium, Gelidibacter, Weeksella, Pedobacter, Sphingobacterium;***Firmicutes**: Bacillus, Paenibacillus, Sporosarcina, Staphylococcus*, Facklamia, Carnobacterium, Jeotgalicoccus, Aerococcus, Desemzia, Trichococcus, Lactococcus, Streptococcus, Dialister, Anaerococcus, Finegoldia, Peptoniphilus;**Proteobacteria:***Devosia, Agrobacterium, Paracoccus, Achromobacter, Acidovorax, Psychrobacter, Pseudomonas, Brevundimonas, Caulobacter, Mycoplana, Phenylobacterium, Ochrobactrum, Methylobacterium, Paracoccus, Rhodobacter, Kaistobacter, Sphingomonas, Comamonas, Neisseria, Erwinia, Acinetobacter, Enhydrobacter, Luteimonas*	**Ascomycota*:*** Aspergillus (A. fumigatus) #, Mycosphaerella, Epicoccum, Preussia Cladophialophora, Penicillium *, Thelebolus, Debaryomyces, Wickerhamomyces, Scopulariopsis, Alternaria#; **Basidiomycota*:*** *Sporobolomyces, Filobasidium, Malassezia*
3	**Actinobacteria**: Brevibacterium, Oerskovia, Corynebacterium, Brachybacterium, Dietzia, Rhodococcus, Streptomyces, Micrococcus *, Curtobacterium, Leucobacter, Pseudoclavibacter, Salinibacterium, Yonghaparkia, Arthrobacter, Aeromicrobium;**Bacteroidetes**: *Myroides, Chryseobacterium, Wautersiella, Weeksella, Pedobacter;***Firmicutes**: Bacillus*, Paenibacillus, Sporosarcina, Staphylococcus *, Facklamia, Carnobacterium, Planomicrobium, Jeotgalicoccus, Aerococcus, Desemzia, Trichococcus, Anaerococcus, Finegoldia, Peptoniphilus;**Proteobacteria:**Devosia, Agrobacterium, Paracoccus, Achromobacter, Tetrathiobacter, Acidovorax, Psychrobacter, Pseudomonas*, Brevundimonas, Caulobacter, Mycoplana, Phenylobacterium, Ochrobactrum, Paracoccus, Sphingomonas, Pigmentiphaga, Tetrathiobacter, Comamonas, Erwinia, Acinetobacter, Luteimonas	**Ascomycota*:*** *Mycosphaerella, Penicillium, Thelebolus, Candida, Debaryomyces, Aspergillus (A. fumigatus) #, Geotrichum #;* **Basidiomycota*:*** *Malassezia*
4	**Actinobacteria**: *Brevibacterium, Oerskovia, Corynebacterium, Brachybacterium, Dietzia, Rhodococcus, Curtobacterium, Leucobacter, Microbacterium, Pseudoclavibacter, Salinibacterium, Yonghaparkia, Arthrobacter, Aeromicrobium, Xylanimicrobium, Sanguibacter;***Bacteroidetes**: *Myroides, Chryseobacterium, Wautersiella, Pedobacter;***Firmicutes**: Bacillus, Brochothrix, Paenibacillus, Sporosarcina, Staphylococcus*, Facklamia, Carnobacterium, Planomicrobium, Jeotgalicoccus, Aerococcus, Desemzia, Trichococcus, Lactococcus;**Proteobacteria:**Devosia, Agrobacterium, Paracoccus, Achromobacter, Tetrathiobacter, Psychrobacter, Pseudomonas, Acinetobacter*, Moellerella#, Brevundimonas, Mycoplana, Phenylobacterium, Ochrobactrum, Pleomorphomonas, Mesorhizobium, Paracoccus, Rhodobacter, Achromobacter, Pigmentiphaga, Erwinia, Acinetobacter, Enhydrobacter, Luteimonas	**Ascomycota*:*** Mycosphaerella, Epicoccum, Penicillium *, Pichia #, Candida, Debaryomyces, Wickerhamomyces *, Scopulariopsis, Aspergillus (A. fumigatus #), Aureobasidium, Peltigera, Saccharomyces, Microascus **Basidiomycota*:*** *Leucosporidium, Malassezia, Rhodotorula#;* **Zygomycota*:*** *Mucor*
5	**Actinobacteria**: *Brevibacterium, Kocuria #, Micrococcus #, Oerskovia, Corynebacterium, Brachybacterium, Dietzia, Rhodococcus, Curtobacterium, Leucobacter, Microbacterium, Salinibacterium, Yonghaparkia, Arthrobacter, Sanguibacter;***Bacteroidetes**: *Chryseobacterium, Sphingobacterium, Wautersiella;***Firmicutes**: Bacillus, Paenibacillus, Sporosarcina, Staphylococcus *, Facklamia, Jeotgalicoccus, Aerococcus, Lactococcus, Helcococcus;**Proteobacteria:***Devosia, Agrobacterium, Paracoccus, Achromobacter, Tetrathiobacter, Acidovorax, Pseudomonas, Brevundimonas, Caulobacter, Mycoplana, Ochrobactrum, Aminobacter, Rhodobacter, Sphingomonas, Achromobacter, Pigmentiphaga, Janthinobacterium, Ralstonia, Acinetobacter, Enhydrobacter, Luteimonas*	**Ascomycota*:*** Penicillium *, Aspergillus #, Alternaria#; **Basidiomycota*:*** *Sampaiozyma*
6	**Actinobacteria**: *Oerskovia, Kocuria #, Micrococcus #, Corynebacterium, Streptomyces, Agromyces, Curtobacterium, Leucobacter, Microbacterium, Pseudoclavibacter, Salinibacterium, Yonghaparkia, Nocardia, Xylanimicrobium, Propionibacterium, Sanguibacter;***Firmicutes**: Bacillus, Paenibacillus, Sporosarcina, Staphylococcus *, Facklamia, Carnobacterium, Paenisporosarcina, Lactococcus;**Proteobacteria:***Achromobacter, Acidovorax, Psychrobacter, Pseudomonas, Sphingomonas, Sphingopyxis*	**Ascomycota*:*** Mycosphaerella, Epicoccum, Alternaria, Penicillium *, Debaryomyces, Scopulariopsis; **Basidiomycota*:*** *Filobasidium. Malassezia*
7	**Actinobacteria**: *Brevibacterium, Oerskovia, Corynebacterium, Brachybacterium, Dietzia, Rhodococcus, Streptomyces, Curtobacterium, Leucobacter, Microbacterium, Yonghaparkia, Arthrobacter, Micrococcus, Nocardiopsis, Propionibacterium, Sanguibacter;***Firmicutes**: Bacillus, Paenibacillus, Sporosarcina, Staphylococcus *, Carnobacterium, Paenisporosarcina;**Proteobacteria:**Devosia, Agrobacterium, Psychrobacter *, Pseudomonas, Brevundimonas, Caulobacter, Mycoplana, Ochrobactrum, Paracoccus, Sphingomonas, Ralstonia	**Ascomycota*:*** *Mycosphaerella, Arachnomyces, Penicillium, Aureobasidium, Alternaria, Meyerozyma, Thelebolus, Scopulariopsis, Candida, Acrostalagmus, Microascus;* **Basidiomycota*:*** *Rhodotorula, Cutaneotrichosporon, Guehomyces, Malassezia, Cryptococcus#, Naganishia #*
8	**Actinobacteria**: *Brevibacterium, Micrococcus #, Oerskovia, Corynebacterium, Brachybacterium, Dietzia, Rhodococcus, Streptomyces, Curtobacterium, Leucobacter, Microbacterium, Yonghaparkia, Arthrobacter, Xylanimicrobium, Propionibacterium, Sanguibacter;***Bacteroidetes**: *Myroides, Chryseobacterium, Wautersiella, Gelidibacterm Weeksella;***Firmicutes**: Bacillus, Paenibacillus, Sporosarcina, Staphylococcus *, Facklamia, Cohnella, Paenisporosarcina, Planomicrobium, Jeotgalicoccus, Desemzia, Trichococcus, Lactococcus;**Proteobacteria:***Devosia, Agrobacterium, Paracoccus, Achromobacter, Tetrathiobacter, Psychrobacter, Pseudomonas, Neisseria (N. flavescens) #, Brevundimonas, Caulobacter, Mycoplana, Balneimonas, Ochrobactrum, Nitratireductor, Paracoccus, Sphingomonas, Sphingopyxis, Methylocaldum, Acinetobacter, Enhydrobacter, Luteimonas*	ns

1—man, 0–10 years old; 2—woman, 0–10 years old; 3—man, 11–17 years old; 4—woman, 11–17 years old; 5—man, 18–50 years old; 6—woman, 18–50 years old; 7—man, >60 years old; 8—woman, >60 years old; ns—not studied in next-generation sequencing method; * identified genus in both culture and next-generation sequencing method; # microorganisms identified exclusively in culture method; may be pathogenic species according to Directive 2000/54/EC of the European Parliament and of the Council of 18 September 2000 on the protection of workers from risks related to exposure to biological agents at work. Off J Eur Communities. L. 262/21 (2000) [28].

**Table 5:** Species present on the human feet skin surface marked out for sanitizing and antimicrobial properties of textile products.

Bacteria	Fungi
*Arthrobacter psychrolactophilus, Bacillus licheniformis, Brachybacterium conglomeratum, Corynebacterium jeikeium, Jeotgalicoccus psychrophilus, Kocuria rhizophila, Micrococcus luteus, Neisseria flavescens, Pseudoclavibacter bifida, Pseudomonas graminis, Pseudomonas oryzihabitans, Psychrobacter marincola, Rhodococcus fascians, Staphylococcus equorum, Staphylococcus haemolyticus, Staphylococcus hominis, Staphylococcus warneri*	**Molds**: *Aspergillus candidus, Aspergillus flavus, Aspergillus fumigatus, Aspergillus niger, Geotrichum candidum, Penicillium citrinum, Penicillium glabrum* **Yeast**: *Cryptococcus magnus, Malassezia restricta, Meyerozyma quilermondii, Naganishia diffluens, Pichia kudriavzevii, Rhodotorula mucilaginosa, Wickerhamomyces anomalus*

## Supplementary Materials

The following are available online at https://www.mdpi.com/1660-4601/16/18/3503/s1, Table S1. Biodiversity of bacteria in the samples—high-throughput sequencing; Table S2. Biodiversity of fungi in the samples—high-throughput sequencing.
