# Supplementary material for: Factors Influencing Microbiological Biodiversity of Human Foot Skin
Source: Int J Environ Res Public Health. 2019 Sep 19;16(18):3503. doi: 10.3390/ijerph16183503 (PMC6765982; doi:10.3390/ijerph16183503)
Supplement: Supplementary file 1 [file ijerph-16-03503-s001.zip › Table S1.docx]

**Table S1. Biodiversity of bacteria in the samples—high-throughput sequencing.**

| **Phylum** | **Genus** | **Relative Abundance (%)** | | | | | | | |
| --- | --- | --- | --- | --- | --- | --- | --- | --- | --- |
|  |  | **1** | **2** | **3** | **4** | **5** | **6** | **7** | **8** |
| **Other (bacteria)** | **Other (bacteria)** | 0.54867 | 0.37815 | 0.57778 | 9.36913 | 1.82978 | 0.77193 | 0.45806 | 0.80404 |
| **Actinobacteria** | ***Actinomyces*** | 0.00000 | 0.01195 | 0.00000 | 0.00000 | 0.00000 | 0.00000 | 0.00000 | 0.00000 |
|  | ***Brevibacterium*** | 0.00741 | 0.03671 | 0.01315 | 0.04800 | 0.01372 | 0.00000 | 3.22379 | 0.27792 |
|  | ***Oerskovia*** | 0.52972 | 0.03585 | 0.06274 | 0.11552 | 0.09789 | 29.96757 | 1.24071 | 0.08565 |
|  | ***Corynebacterium*** | 0.73815 | 10.72642 | 3.78848 | 0.02847 | 4.33291 | 0.02154 | 0.01658 | 0.05768 |
|  | ***Corynebacterium durum*** | 0.00000 | 0.01110 | 0.00000 | 0.00000 | 0.00000 | 0.00000 | 0.00000 | 0.00000 |
|  | ***Brachybacterium*** | 2.28366 | 0.05378 | 0.01821 | 0.20175 | 0.02562 | 0.00000 | 0.20613 | 0.22461 |
|  | ***Brachybacterium conglomeratum*** | 0.02224 | 0.00000 | 0.00304 | 0.00651 | 0.00549 | 0.00000 | 0.00474 | 0.00000 |
|  | ***Dietzia*** | 0.00412 | 0.05122 | 0.02024 | 2.02888 | 0.00915 | 0.00000 | 0.08766 | 0.93950 |
|  | ***Microbacteriaceae* (family)** | 23.66457 | 0.14597 | 0.07893 | 0.15375 | 0.00457 | 6.47582 | 0.05449 | 0.22723 |
|  | ***Agromyces*** | 0.00000 | 0.00000 | 0.00000 | 0.00000 | 0.00000 | 0.14242 | 0.00000 | 0.00000 |
|  | ***Curtobacterium*** | 0.73321 | 0.00512 | 0.00607 | 0.00325 | 0.00091 | 0.03710 | 0.00237 | 0.00175 |
|  | ***Leucobacter*** | 0.04366 | 0.04866 | 0.01214 | 0.21802 | 0.02287 | 0.00479 | 0.01422 | 0.56807 |
|  | ***Microbacterium*** | 0.02060 | 0.00085 | 0.00000 | 0.00000 | 0.00000 | 0.00000 | 0.00000 | 0.00699 |
|  | ***Microbacterium*** | 0.01236 | 0.00085 | 0.00000 | 0.00244 | 0.03111 | 0.11250 | 0.12794 | 0.00000 |
|  | ***Pseudoclavibacter bifida*** | 0.00906 | 0.00512 | 0.00101 | 0.00325 | 0.00000 | 0.00838 | 0.00000 | 0.00000 |
|  | ***Salinibacterium*** | 0.01236 | 0.00000 | 0.00405 | 0.39536 | 0.01830 | 0.00239 | 0.00000 | 0.00000 |
|  | ***Yonghaparkia*** | 0.03872 | 0.04353 | 0.00506 | 0.01546 | 0.00457 | 0.98735 | 0.01816 | 0.00612 |
|  | ***Micrococcaceae* (family)** | 2.60578 | 0.64618 | 1.22235 | 1.84584 | 1.87918 | 0.00120 | 0.03080 | 0.37755 |
|  | ***Arthrobacter*** | 0.50830 | 0.11950 | 0.10928 | 0.04881 | 0.34766 | 0.00000 | 0.29774 | 0.14333 |
|  | ***Arthrobacter psychrolactophilus*** | 0.00000 | 0.12036 | 0.03542 | 0.40187 | 0.63585 | 0.00000 | 0.00000 | 0.00612 |
|  | ***Micrococcus*** | 0.10380 | 0.00085 | 0.00101 | 0.00000 | 0.00000 | 0.00000 | 0.00079 | 0.00000 |
|  | ***Mycobacterium*** | 0.00000 | 0.01195 | 0.00000 | 0.00000 | 0.00000 | 0.00000 | 0.00000 | 0.00000 |
|  | ***Nocardia*** | 0.00000 | 0.00000 | 0.00000 | 0.00000 | 0.00000 | 0.04428 | 0.00000 | 0.00000 |
|  | ***Rhodococcus*** | 0.03131 | 0.11268 | 0.24690 | 1.27476 | 0.31198 | 0.00000 | 0.35776 | 0.03583 |
|  | ***Rhodococcus fascians*** | 0.05849 | 0.07768 | 0.02327 | 0.40268 | 0.00091 | 0.00000 | 0.00079 | 0.00000 |
|  | ***Aeromicrobium*** | 0.00000 | 0.00000 | 0.00101 | 0.00976 | 0.00000 | 0.00000 | 0.00000 | 0.00000 |
|  | ***Nocardioides*** | 0.00000 | 0.01536 | 0.00000 | 0.00000 | 0.00000 | 0.00000 | 0.00000 | 0.00000 |
|  | ***Nocardiopsis exhalans*** | 0.00000 | 0.05548 | 0.00000 | 0.00000 | 0.00000 | 0.00000 | 0.27484 | 0.00000 |
|  | ***Xylanimicrobium*** | 0.00000 | 0.00000 | 0.00000 | 0.00081 | 0.00000 | 0.01077 | 0.00000 | 0.00350 |
|  | ***Propionibacterium acnes*** | 0.00000 | 0.01878 | 0.00000 | 0.00000 | 0.00000 | 0.00479 | 0.00079 | 0.00175 |
|  | ***Sanguibacter*** | 0.00412 | 0.00000 | 0.00000 | 0.00407 | 0.00183 | 0.05386 | 0.07819 | 0.00087 |
|  | ***Streptomyces*** | 0.04531 | 0.02475 | 0.00202 | 0.00000 | 0.00000 | 25.83267 | 0.00079 | 0.00087 |
| **Bacteroidetes** | ***Gelidibacter*** | 0.00000 | 0.01963 | 0.00000 | 0.00000 | 0.00000 | 0.00000 | 0.00000 | 0.53224 |
|  | ***Myroides*** | 0.00000 | 0.00939 | 0.00607 | 20.24893 | 0.00000 | 0.00000 | 0.00000 | 0.00874 |
|  | ***Weeksellaceae* (family)** | 0.00165 | 0.41656 | 1.51782 | 13.71731 | 0.40987 | 0.00000 | 0.00000 | 0.00000 |
|  | ***Chryseobacterium*** | 0.00247 | 0.04353 | 0.22059 | 1.00061 | 1.02284 | 0.00000 | 0.00000 | 0.00437 |
|  | ***Wautersiella*** | 0.00000 | 0.00000 | 0.00101 | 0.09437 | 0.00091 | 0.00000 | 0.00000 | 7.01351 |
|  | ***Weeksella*** | 0.00000 | 0.06061 | 0.69213 | 0.00000 | 0.00000 | 0.00000 | 0.00000 | 0.00175 |
|  | ***Sphingobacteriaceae* (family)** | 0.00330 | 1.79855 | 16.31049 | 6.80903 | 0.00000 | 5.46094 | 0.00158 | 0.78569 |
|  | ***Pedobacter*** | 0.00000 | 0.01622 | 0.05464 | 0.14399 | 0.00000 | 0.00000 | 0.00000 | 0.00000 |
|  | ***Sphingobacterium*** | 0.00000 | 0.00512 | 0.00000 | 0.00000 | 0.92678 | 0.00000 | 0.00000 | 0.00000 |
| **Chloroflexi** | ***Thermomicrobia* (class)** | 0.00000 | 0.02049 | 0.03238 | 0.32540 | 0.04483 | 0.00000 | 0.01343 | 0.73238 |
| **Cyanobacteria** | ***Chloroplast* (class)** | 0.00000 | 0.07768 | 0.00101 | 0.00000 | 0.00000 | 0.56369 | 0.00000 | 0.00000 |
| **Firmicutes** | ***Bacillus*** | 0.02389 | 0.12548 | 0.00101 | 0.00488 | 0.00457 | 0.10412 | 1.87489 | 0.01224 |
|  | ***Brochothrix*** | 4.15788 | 0.00000 | 0.00000 | 0.00081 | 0.00000 | 0.00000 | 0.00000 | 0.00000 |
|  | ***Cohnella*** | 0.00000 | 0.00000 | 0.00000 | 0.00000 | 0.00000 | 0.00000 | 0.00000 | 0.00961 |
|  | ***Paenibacillus*** | 0.23397 | 0.00598 | 0.01012 | 1.70917 | 0.10064 | 2.40195 | 2.14972 | 0.42737 |
|  | ***Paenisporosarcina*** | 0.01236 | 0.00000 | 0.00000 | 0.00000 | 0.00000 | 0.01197 | 0.01737 | 0.00175 |
|  | ***Planomicrobium*** | 0.00000 | 0.00000 | 0.00101 | 0.17653 | 0.00000 | 0.00000 | 0.00000 | 0.18528 |
|  | ***Sporosarcina*** | 0.00000 | 0.00000 | 0.00000 | 0.00000 | 0.00000 | 0.00000 | 0.03159 | 0.00000 |
|  | ***Sporosarcina*** | 2.71205 | 0.08365 | 0.03643 | 0.05369 | 0.59376 | 23.84121 | 15.08912 | 0.65285 |
|  | ***Jeotgalicoccus*** | 0.00000 | 0.00000 | 0.00000 | 0.00000 | 0.20768 | 0.00000 | 0.00000 | 0.00000 |
|  | ***Jeotgalicoccus psychrophilus*** | 0.00000 | 0.00768 | 0.00304 | 0.01302 | 0.00549 | 0.00000 | 0.00000 | 0.02447 |
|  | ***Staphylococcus*** | 0.73898 | 46.00683 | 1.09890 | 0.10901 | 0.04117 | 0.02872 | 2.00836 | 1.28210 |
|  | ***Staphylococcus equorum*** | 0.01236 | 0.00256 | 0.00101 | 0.00163 | 0.00000 | 0.00000 | 0.07819 | 0.00787 |
|  | ***Staphylococcus sciuri*** | 0.00000 | 0.00000 | 0.00000 | 0.00244 | 0.00000 | 0.00000 | 0.00079 | 0.70091 |
|  | ***Exiguobacterium*** | 0.93175 | 0.00000 | 0.00000 | 0.00000 | 0.00000 | 0.00000 | 0.00000 | 0.00000 |
|  | ***Aerococcus*** | 0.01318 | 0.03329 | 0.03036 | 0.00895 | 0.00091 | 0.00000 | 0.00000 | 0.00000 |
|  | ***Facklamia*** | 0.00082 | 0.13060 | 0.04655 | 0.16189 | 2.47111 | 0.00359 | 0.00000 | 0.00000 |
|  | ***Carnobacterium*** | 0.00082 | 1.13188 | 0.46445 | 1.26419 | 0.00000 | 0.00239 | 1.49027 | 0.04719 |
|  | ***Desemzia*** | 0.00000 | 0.02219 | 0.00405 | 0.15294 | 0.00000 | 0.00000 | 0.00000 | 0.11187 |
|  | ***Trichococcus*** | 0.00000 | 0.00939 | 0.00304 | 0.00244 | 0.00000 | 0.00000 | 0.00000 | 0.00350 |
|  | ***Lactococcus*** | 0.00000 | 0.01024 | 0.00000 | 0.00081 | 0.00091 | 0.00120 | 0.00000 | 0.00262 |
|  | ***Streptococcus*** | 0.00000 | 0.08621 | 0.00000 | 0.00000 | 0.00000 | 0.00000 | 0.00000 | 0.00000 |
|  | ***Dialister*** | 0.00000 | 0.01451 | 0.00000 | 0.00000 | 0.00000 | 0.00000 | 0.00000 | 0.00000 |
|  | ***Anaerococcus*** | 0.00000 | 0.07000 | 0.01012 | 0.00000 | 0.00000 | 0.00000 | 0.00000 | 0.00000 |
|  | ***Finegoldia*** | 0.00000 | 0.00854 | 0.00101 | 0.00000 | 0.00000 | 0.00000 | 0.00000 | 0.00000 |
|  | ***Helcococcus*** | 0.00082 | 0.00000 | 0.00000 | 0.00000 | 0.22049 | 0.00000 | 0.00000 | 0.00000 |
|  | ***Peptoniphilus*** | 0.00000 | 0.03329 | 0.00101 | 0.00000 | 0.00000 | 0.00000 | 0.00000 | 0.00000 |
| ***Planctomycetes*** | ***Pirellulaceae* (family)** | 0.00000 | 0.00000 | 0.00000 | 0.03579 | 0.00000 | 0.00000 | 0.00000 | 0.28316 |
| **Proteobacteria** | ***Caulobacteraceae* (family)** | 0.00412 | 0.00512 | 0.12244 | 0.07566 | 0.08143 | 0.00120 | 0.01106 | 0.01224 |
|  | ***Caulobacteraceae* (family)** | 5.39857 | 7.95220 | 19.23785 | 10.22168 | 51.70123 | 3.05420 | 41.22302 | 49.60497 |
|  | ***Brevundimonas diminuta*** | 0.00000 | 0.13914 | 0.30964 | 0.10413 | 0.31198 | 0.00000 | 0.07108 | 0.45446 |
|  | ***Caulobacter*** | 0.00000 | 0.00085 | 0.04452 | 0.00000 | 0.00274 | 0.00000 | 0.00158 | 0.00087 |
|  | ***Mycoplana*** | 0.00000 | 0.00598 | 0.00911 | 0.00244 | 0.01006 | 0.00000 | 0.01185 | 0.01049 |
|  | ***Phenylobacterium*** | 0.00082 | 0.16304 | 0.21148 | 0.00081 | 0.00000 | 0.00000 | 0.00000 | 0.00000 |
|  | ***Balneimonas*** | 0.00000 | 0.00000 | 0.00000 | 0.00000 | 0.00000 | 0.00000 | 0.00000 | 0.01486 |
|  | ***Ochrobactrum*** | 0.00000 | 0.00854 | 0.00101 | 0.00976 | 0.66512 | 0.00000 | 0.00158 | 0.00087 |
|  | ***Devosia*** | 0.37237 | 2.02646 | 2.80898 | 7.03681 | 1.59373 | 0.00000 | 0.06318 | 1.28734 |
|  | ***Methylobacterium adhaesivum*** | 0.00000 | 0.02390 | 0.00000 | 0.00000 | 0.00000 | 0.00000 | 0.00000 | 0.00000 |
|  | ***Pleomorphomonas*** | 0.00000 | 0.00000 | 0.00000 | 0.01464 | 0.00000 | 0.00000 | 0.00000 | 0.00000 |
|  | ***Aminobacter*** | 0.00000 | 0.00000 | 0.00000 | 0.00000 | 0.00915 | 0.00000 | 0.00000 | 0.00000 |
|  | ***Mesorhizobium*** | 0.00000 | 0.00000 | 0.00000 | 0.01302 | 0.00000 | 0.00000 | 0.00000 | 0.00000 |
|  | ***Nitratireductor*** | 0.00000 | 0.00000 | 0.00000 | 0.00000 | 0.00000 | 0.00000 | 0.00000 | 0.03321 |
|  | ***Agrobacterium*** | 2.18974 | 0.07512 | 0.19428 | 0.06345 | 0.01006 | 0.00000 | 0.00553 | 0.01311 |
|  | ***Paracoccus*** | 0.00082 | 0.01622 | 0.00101 | 0.04556 | 0.00000 | 0.00000 | 0.00079 | 0.00350 |
|  | ***Paracoccus marcusii*** | 3.96675 | 0.06402 | 0.01923 | 0.15538 | 0.03202 | 0.00000 | 0.00000 | 0.19489 |
|  | ***Rhodobacter*** | 0.00000 | 0.01536 | 0.00000 | 0.02359 | 0.00274 | 0.00000 | 0.00000 | 0.00000 |
|  | ***Kaistobacter*** | 0.00000 | 0.01366 | 0.00000 | 0.00000 | 0.00000 | 0.00000 | 0.00000 | 0.00000 |
|  | ***Sphingomonas*** | 0.00000 | 0.03158 | 0.00101 | 0.00000 | 0.00091 | 0.00120 | 0.00079 | 0.02010 |
|  | ***Sphingopyxis alaskensis*** | 0.00000 | 0.00000 | 0.00000 | 0.00000 | 0.00000 | 0.00239 | 0.00000 | 0.00699 |
|  | ***Alcaligenaceae* (family)** | 0.00906 | 0.41485 | 1.60889 | 1.71405 | 0.05672 | 0.00000 | 0.00000 | 0.23772 |
|  | ***Achromobacter*** | 0.87656 | 0.21255 | 0.00101 | 0.14155 | 0.27996 | 0.00120 | 0.00000 | 11.38068 |
|  | ***Pigmentiphaga*** | 0.18042 | 0.00000 | 0.00911 | 0.07077 | 0.09515 | 0.00000 | 0.00000 | 0.00000 |
|  | ***Tetrathiobacter kashmirensis*** | 0.00000 | 0.00000 | 0.00405 | 0.07484 | 1.89931 | 0.00000 | 0.00000 | 0.14246 |
|  | ***Comamonadaceae* (family)** | 0.03378 | 0.51216 | 0.79433 | 0.04637 | 17.01234 | 0.00239 | 0.00000 | 0.00262 |
|  | ***Acidovorax*** | 0.00000 | 0.00000 | 0.00000 | 0.00000 | 0.01464 | 0.00000 | 0.00000 | 0.00000 |
|  | ***Acidovorax facilis*** | 0.00082 | 0.05975 | 0.14268 | 0.00000 | 3.02553 | 0.00239 | 0.00000 | 0.00000 |
|  | ***Comamonas*** | 0.00000 | 0.11780 | 0.09815 | 0.00000 | 0.00000 | 0.00000 | 0.00000 | 0.00000 |
|  | ***Janthinobacterium*** | 0.00000 | 0.00000 | 0.00000 | 0.00000 | 0.00915 | 0.00000 | 0.00000 | 0.00000 |
|  | ***Ralstonia*** | 0.00906 | 0.00000 | 0.00000 | 0.00000 | 0.00732 | 0.00000 | 0.00553 | 0.00000 |
|  | ***Neisseria*** | 0.00000 | 0.02902 | 0.00000 | 0.00000 | 0.00000 | 0.00000 | 0.00000 | 0.00000 |
|  | ***Erwinia*** | 0.00000 | 0.01024 | 0.02631 | 0.44011 | 0.00000 | 0.00000 | 0.00000 | 0.00000 |
|  | ***Methylocaldum*** | 0.00000 | 0.00000 | 0.00000 | 0.00000 | 0.00000 | 0.00000 | 0.00000 | 0.03059 |
|  | ***Moraxellaceae* (family)** | 1.72263 | 0.09987 | 0.14773 | 0.65406 | 0.00091 | 0.00000 | 27.17954 | 2.10012 |
|  | ***Acinetobacter*** | 0.00082 | 0.35681 | 0.50189 | 0.23266 | 0.09149 | 0.00000 | 0.00000 | 0.76996 |
|  | ***Acinetobacter johnsonii*** | 0.00000 | 0.00512 | 0.00202 | 0.00000 | 0.91397 | 0.00000 | 0.00000 | 0.10051 |
|  | ***Acinetobacter lwoffii*** | 0.00000 | 0.01451 | 0.06071 | 0.09925 | 0.00000 | 0.00000 | 0.00000 | 0.00000 |
|  | ***Enhydrobacter*** | 0.00000 | 0.04353 | 0.00000 | 0.00081 | 0.00091 | 0.00000 | 0.00000 | 0.00787 |
|  | ***Psychrobacter*** | 36.53365 | 0.43961 | 0.35922 | 8.31971 | 0.00000 | 0.00120 | 1.49027 | 10.09421 |
|  | ***Psychrobacter marincola*** | 0.01483 | 0.00256 | 0.00202 | 0.01952 | 0.00000 | 0.00000 | 0.02132 | 0.00262 |
|  | ***Psychrobacter pacificensis*** | 0.00165 | 0.50704 | 0.15279 | 1.66524 | 0.00091 | 0.00000 | 0.55678 | 1.10031 |
|  | ***Psychrobacter sanguinis*** | 0.00000 | 0.03073 | 0.02530 | 0.00163 | 0.00000 | 0.00000 | 0.00000 | 0.00699 |
|  | ***Pseudomonas*** | 0.00824 | 0.43534 | 1.10801 | 4.28635 | 4.55706 | 0.00359 | 0.00000 | 0.67295 |
|  | ***Pseudomonas balearica*** | 0.00000 | 0.00000 | 0.02530 | 0.00000 | 0.00000 | 0.00000 | 0.00000 | 0.00000 |
|  | ***Pseudomonas fragi*** | 0.01153 | 0.00000 | 0.00000 | 0.04230 | 0.00000 | 0.00000 | 0.00000 | 0.05069 |
|  | ***Pseudomonas viridiflava*** | 0.04531 | 12.34913 | 22.16724 | 0.23754 | 0.26349 | 0.00239 | 0.00079 | 4.02021 |
|  | ***Xanthomonadaceae* (family)** | 7.74649 | 10.20913 | 22.30688 | 1.46838 | 1.69529 | 0.00000 | 0.02764 | 0.96310 |
|  | ***Luteimonas*** | 0.00000 | 0.33461 | 0.53933 | 0.06671 | 0.02104 | 0.00000 | 0.00000 | 0.05768 |

1—man. 0–10 years old; 2—woman. 0–10 years old; 3—man. 11–17 years old; 4—woman. 11–17 years old; 5—man. 18–50 years old; 6—woman. 18–50 years old; 7—man. >60 years old; 8—woman. >60 years old.
