# Supplementary material for: Factors Influencing Microbiological Biodiversity of Human Foot Skin
Source: Int J Environ Res Public Health. 2019 Sep 19;16(18):3503. doi: 10.3390/ijerph16183503 (PMC6765982; doi:10.3390/ijerph16183503)
Supplement: Supplementary file 1 [file ijerph-16-03503-s001.zip › Table S2 Biodiveristy of fungi in the samples.docx]

**Table S2. Biodiversity of fungi in the samples—high-throughput sequencing**

| **Phylum** | **Genus** | **Relative Abundance [%]** | | | | | | |
| --- | --- | --- | --- | --- | --- | --- | --- | --- |
|  |  | **1** | **2** | **3** | **4** | **5** | **6** | **7** |
| **Ascomycota** | ***Cladosporium grevilleae*** | 0.00000 | 0.00000 | 0.00000 | 0.00000 | 0.00000 | 0.00000 | 0.09035 |
|  | ***Mycosphaerella tassiana*** | 0.00141 | 17.84187 | 0.02863 | 0.81407 | 0.00000 | 0.01282 | 0.27548 |
|  | ***Capnodiales* sp. (order)** | 0.00282 | 19.20864 | 0.00000 | 0.38191 | 3.19410 | 56.65873 | 0.38360 |
|  | ***Aureobasidium pullulans*** | 0.00000 | 0.00000 | 0.00000 | 0.04020 | 0.00000 | 0.00000 | 0.08294 |
|  | ***Alternaria rosae*** | 0.00000 | 0.00000 | 0.00000 | 0.00000 | 0.00000 | 0.05522 | 0.05332 |
|  | ***Epicoccum nigrum*** | 0.00000 | 1.35540 | 0.00000 | 0.15075 | 0.00000 | 0.00148 | 0.00000 |
|  | ***Preussia persica*** | 0.00000 | 0.26568 | 0.00000 | 0.00000 | 0.00000 | 0.00000 | 0.00000 |
|  | ***Arachnomyces nodosetosus*** | 0.00000 | 0.00000 | 0.00000 | 0.00000 | 0.00000 | 0.00000 | 1.92541 |
|  | ***Cladophialophora* sp.** | 0.00000 | 3.16829 | 0.00000 | 0.00000 | 0.00000 | 0.00000 | 0.00000 |
|  | ***Penicillium aurantiogriseum*** | 87.50141 | 0.00000 | 0.28629 | 25.04523 | 0.00000 | 0.00000 | 18.72982 |
|  | ***Penicillium cinnamopurpureum*** | 0.00000 | 0.00000 | 0.00000 | 0.14070 | 0.00000 | 0.00000 | 0.00000 |
|  | ***Penicillium corylophilum*** | 0.00565 | 1.61966 | 0.02863 | 0.01005 | 12.53071 | 0.00049 | 0.07850 |
|  | ***Penicillium magnielliptisporum*** | 0.05507 | 0.00000 | 0.00000 | 0.00000 | 0.00000 | 0.00000 | 0.00000 |
|  | ***Penicillium polonicum*** | 0.01977 | 0.00000 | 0.00000 | 0.06030 | 0.00000 | 0.00000 | 0.07109 |
|  | ***Sagenomella griseoviridis*** | 0.58744 | 0.00000 | 0.00000 | 0.00000 | 0.00000 | 0.00000 | 0.00000 |
|  | ***Peltigera monticola*** | 0.00000 | 0.00000 | 0.00000 | 0.13065 | 0.00000 | 0.00000 | 0.00000 |
|  | ***Helotiaceae* sp. (family)** | 0.00000 | 0.00000 | 0.00000 | 0.00000 | 0.00000 | 0.00000 | 0.31399 |
|  | ***Thelebolus globosus*** | 0.00000 | 0.00142 | 0.02863 | 0.00000 | 0.00000 | 0.00000 | 28.92266 |
|  | ***Leotiomycetes* sp. (class)** | 0.00000 | 0.00000 | 0.14314 | 0.22111 | 0.00000 | 0.00000 | 3.20507 |
|  | ***Meyerozyma guilliermondii*** | 0.00000 | 0.00000 | 0.00000 | 0.00000 | 0.00000 | 0.00000 | 0.23549 |
|  | ***Saccharomyces cerevisiae*** | 0.00000 | 0.00000 | 0.00000 | 0.27136 | 0.00000 | 0.00000 | 0.00000 |
|  | ***Saccharomycetaceae* sp. (family)** | 0.00000 | 0.00000 | 0.00000 | 0.17085 | 0.00000 | 0.00000 | 0.00000 |
|  | ***Candida* sp.** | 0.00000 | 0.00000 | 0.40080 | 3.34673 | 0.00000 | 0.00000 | 0.00000 |
|  | ***Candida zeylanoides*** | 0.00000 | 0.00000 | 0.00000 | 0.00000 | 0.00000 | 0.00000 | 0.03258 |
|  | ***Debaryomyces hansenii*** | 0.00000 | 0.01563 | 0.05726 | 16.31156 | 0.00000 | 1.70209 | 0.00000 |
|  | ***Wickerhamomyces anomalus*** | 0.00000 | 0.00000 | 0.00000 | 0.20101 | 0.00000 | 0.00000 | 0.00000 |
|  | ***Wickerhamomyces onychis*** | 0.00000 | 1.01016 | 0.00000 | 0.01005 | 0.00000 | 0.00000 | 0.00000 |
|  | ***Acrostalagmus luteoalbus*** | 0.00000 | 0.00000 | 0.00000 | 0.00000 | 0.00000 | 0.00000 | 0.12293 |
|  | ***Nectriaceae* sp. (family)** | 0.00000 | 0.00000 | 0.00000 | 0.00000 | 3.93120 | 0.16469 | 0.00296 |
|  | ***Microascus brevicaulis*** | 0.00000 | 0.00000 | 0.00000 | 0.20101 | 0.00000 | 0.00000 | 0.00148 |
|  | ***Scopulariopsis candida*** | 0.00000 | 0.00284 | 0.00000 | 0.15075 | 0.00000 | 0.00049 | 23.00868 |
|  | ***Microascaceae* sp. (family)** | 0.00000 | 0.00000 | 0.11451 | 0.06030 | 0.00000 | 0.00000 | 0.00000 |
|  | ***Apiosporaceae* sp. (family)** | 0.00000 | 2.24480 | 0.00000 | 0.00000 | 0.00000 | 0.00000 | 0.00000 |
|  | ***Sordariomycetes* sp. (class)** | 0.49706 | 0.00000 | 0.00000 | 0.00000 | 0.00000 | 0.00000 | 0.00000 |
|  | ***Ascomycota* sp. (phylum)** | 0.00000 | 2.53321 | 0.00000 | 0.12060 | 0.00000 | 0.00000 | 1.52552 |
| **Basidiomycota** | ***Laetiporus* sp.** | 1.06332 | 0.00000 | 0.00000 | 0.00000 | 0.00000 | 0.00000 | 0.00000 |
|  | ***Agaricomycetes* sp. (class)** | 0.00000 | 2.42665 | 0.00000 | 0.00000 | 0.00000 | 0.00000 | 0.00000 |
|  | ***Leucosporidium* sp.** | 0.00000 | 0.00000 | 0.00000 | 28.35176 | 0.00000 | 0.00000 | 0.00000 |
|  | ***Sampaiozyma ingeniosa*** | 0.00000 | 0.00000 | 0.00000 | 0.00000 | 3.68550 | 0.00000 | 0.00000 |
|  | ***Rhodotorula mucilaginosa*** | 0.00000 | 0.00000 | 0.00000 | 0.00000 | 0.00000 | 0.00000 | 17.65159 |
|  | ***Sporobolomyces roseus*** | 0.00000 | 3.95965 | 0.00000 | 0.00000 | 0.00000 | 0.00000 | 0.00000 |
|  | ***Guehomyces pullulans*** | 0.00000 | 0.00000 | 0.00000 | 0.00000 | 0.00000 | 0.00000 | 0.12589 |
|  | ***Filobasidium floriforme*** | 0.00000 | 0.00000 | 0.00000 | 0.00000 | 0.00000 | 0.00690 | 0.00000 |
|  | ***Filobasidium magnum*** | 0.00000 | 2.47496 | 0.00000 | 0.00000 | 0.00000 | 0.00000 | 0.00000 |
|  | ***Goffeauzyma gastrica*** | 3.04875 | 0.00000 | 0.00000 | 0.00000 | 0.00000 | 0.00000 | 0.00000 |
|  | ***Cutaneotrichosporon jirovecii*** | 0.00000 | 0.00000 | 0.00000 | 0.00000 | 0.00000 | 0.00000 | 1.84099 |
|  | ***Malassezia cuniculi*** | 0.70041 | 0.00000 | 0.00000 | 0.02010 | 0.00000 | 0.00000 | 0.00000 |
|  | ***Malassezia globosa*** | 0.00000 | 6.01264 | 0.02863 | 0.00000 | 0.00000 | 0.00000 | 0.00000 |
|  | ***Malassezia restricta*** | 0.00000 | 0.20601 | 0.14314 | 0.14070 | 0.00000 | 0.00197 | 0.06961 |
|  | ***Malassezia sympodialis*** | 0.00000 | 1.43070 | 0.05726 | 0.00000 | 0.00000 | 0.00000 | 0.00000 |
|  | ***Basidiomycota* sp. (phylum)** | 1.61828 | 0.00000 | 0.00000 | 0.00000 | 0.00000 | 0.00000 | 0.15107 |
| **Zygomycota** | ***Mucor mucedo*** | 0.00000 | 0.00000 | 0.00000 | 1.23618 | 0.00000 | 0.00000 | 0.00000 |
| **Unidentified (Fungi)** | **Unidentified (Fungi)** | 2.57993 | 28.81864 | 54.25136 | 1.29648 | 3.68550 | 41.34531 | 0.70648 |

1—man. 0–10 years old; 2—woman. 0–10 years old; 3—man. 11–17 years old; 4—woman. 11–17 years old; 5—man. 18–50 years old; 6—woman. 18–50 years old; 7—man. >60 years old.
